# A novel human *CRYGD* mutation in a juvenile autosomal dominant cataract

**Published:** 2010-05-22

**Authors:** Mascarenhas Roshan, Pai H. Vijaya, G. Rao Lavanya, Prasada K. Shama, S.T. Santhiya, Jochen Graw, P.M. Gopinath, K. Satyamoorthy

**Affiliations:** 1 Department of Biotechnology, Manipal Life Sciences Centre, Manipal University, Manipal, India; 2 Department of Ophthalmology, Kasturba Hospital, Manipal University, Manipal, India; 3 Department of Genetics, Dr. ALM P.G. Institute of Basic Medical Sciences, University of Madras, Taramani, Chennai, India; 4 Institute of Developmental Genetics, Helmholtz Center Munich, German Research Center for Environmental Health, Neuherberg, Germany

## Abstract

**Purpose:**

Identification of causal mutation in the crystallin, connexin, and paired box gene 6 (*PAX6*) genes associated with childhood cataract in patients from India.

**Methods:**

In this study, forty eight members from seventeen families and 148 sporadic cases of childhood cataract were evaluated. Clinical and ophthalmologic examinations were performed on available affected and unaffected family members. Samples of genomic DNA were PCR amplified to screen for mutations in the candidate genes viz., alpha-A crystallin (*CRYAA*), beta- B2 crystallin (*CRYBB2*), gamma-A crystallin (*CRYGA*), gamma-B crystallin (*CRYGB*), gamma-C crystallin (*CRYGC*), gamma-D crystallin (*CRYGD*), gap junction alpha-3 (*GJA3*), gap junction alpha-8 (*GJA8*), and *PAX6* based on polymerase chain reaction and single strand conformation polymorphism (PCR-SSCP) analysis. Samples showing any band mobility shift were subjected to bidirectional sequencing to confirm the variation. Co-segregation of the observed change with the disease phenotype was further tested by restriction fragment length polymorphism (RFLP) for the appropriate restriction site.

**Results:**

DNA sequencing analysis of *CRYAA, CRYBB2*, *CRYGA-D*, *GJA3, GJA8,* and *PAX6* of the affected members of a family (C-35) showed a novel heterozygous missense mutation C>A at position 229 in *CRYGD* in three affected members of family C-35 with anterior polar coronary cataract. This variation C229A substitution created a novel restriction site for AluI and resulted in a substitution of highly conserved arginine at position 77 by serine (R77S). AluI restriction site analysis confirmed the transversion mutation. Analysis of the available unaffected members of the family (C-35) and 100 unrelated control subjects (200 chromosomes) of the same ethnic background did not show R77S variation. Data generated using ProtScale and PyMOL programs revealed that the mutation altered the stability and solvent-accessibility of the CRYGD protein.

**Conclusions:**

We describe here a family having anterior polar coronary cataract that co-segregates with the novel allele R77S of *CRYGD* in all the affected members. The same was found to be absent in the ethnically matched controls (n=100) studied. Interestingly the residue Arg has been frequently implicated in four missense (R15C, R15S, R37S, and R59H) and in one truncation mutation (R140X) of *CRYGD*. In two of the reported mutations Arg residues have been replaced with Serine. This finding further expands the mutation spectrum of *CRYGD* in association with childhood cataract and demonstrates a possible mechanism of cataractogenesis. Screening of other familial (n=48) and sporadic (n=148) cases of childhood cataract, did not reveal any previously reported or novel mutation in the candidate genes screened.

## Introduction

Childhood cataract is the most common form of treatable blindness in children [1]. It accounts for more than 1 million blind children in Asia and about 10% of childhood blindness worldwide [2]. Non-syndromic congenital cataracts have an estimated incidence of 1–6 per 10,000 live births [3,4]. The prevalence of childhood cataract varies between different population, geographic area, socioeconomic condition and other factors. Etiologic factors include genetic, metabolic, systemic disorders, intrauterine infections, and trauma. Approximately 50% of the childhood cataracts are genetic and at least one-third may have the familial basis. It is both clinically and genetically diverse as demonstrated by several investigators suggesting involvement of possible modifying factors [2]. Hereditary cataracts typically follow Mendelian inheritance with majority being autosomal dominant type with complete penetrance and may be either static or progressive. Cataracts are classified based on morphology, location, or time of onset. Childhood cataracts have been mapped to 36 genetic loci and mutations in 22 genes have been identified toward the pathogenesis of various forms of congenital and developmental cataract including 12 loci for autosomal recessive cataract [5-8].

Biochemically up to 90% of the water soluble proteins in the vertebrate lens belong to α-, β-, and γ-crystallins. Being a part of small heat shock protein the α-crystallins form high-molecular aggregates and function as molecular chaperones. The β/γ-crystallin superfamily exhibits a characteristic Greek key motif, in a quadruple organization showing two in the NH_2-_ and two in the COOH-terminal domain. Evolutionary analysis has demonstrated the relationship of crystallins to other stress proteins and is expressed in other tissues of the body as well [9].

The human gamma-crystallins (*CRYG*) gene cluster comprise six genes including two pseudogenes. The spectrum of mutations in *CRYG* gene leading to diverse cataract phenotypes is on the increase [10,11]. The aim of the present study therefore was to screen families with inherited cataracts to document known as well as novel mutations if any in human genes coding for alpha-A crystallin (*CRYAA*), beta-B2 crystallin (*CRYBB2*), gamma-crystallins (*CRYG*), gap junction alpha-3 and alpha-8 (*GJA3*, *GJA8*), and transcription factor paired box gene 6 (*PAX6*).

## Methods

Cases of childhood cataract were registered through Kasturba Hospital (KH), Manipal, India. Clinical details of the proband were recorded in all the cases. Ophthalmic investigation included slit lamp examination with dilated pupils, visual acuity testing, intraocular pressure measurement, and fundus examination done by a senior ophthalmologist (V.P.). In familial cases, identification of cataract phenotype and detailed examination of the affected as well as available unaffected family members were performed. A detailed pedigree of the kindred was ascertained by interviewing the parents or any available family member. Clinical details of the patients who previously had cataract extraction were obtained through medical records. Cases presenting conditions such as unilateral, congenital rubella, systemic disorders, traumatic, syndromic, and other known causes were excluded for further study. Seeking of informed consent from all participants and parents of the probands was in accordance with the Declaration of Helsinki and Institutional Ethical Committee of Manipal University. Blood samples were collected from available affected/unaffected members of the family.

### PCR and single stranded conformational polymorphism (SSCP) analysis

Genomic DNA was extracted from peripheral blood leukocytes using phenol chloroform method [12]. Exons and exon-intron boundaries of *CRYAA*, *CRYBB2*, *CRYGA* >*D, GJA3, GJA8*, and *PAX6* were PCR amplified as per earlier reports on *CRYAA* [13], *CRYBB2, GJA8* [14], *CRYGA>D* [15], *GJA3* [16] and *PAX6* [17].  The PCR reactions were performed at 94 °C for 4 min followed by 29 cycles at 94 °C for 30 s, at optimum annealing temperature for 45 s, at 72 °C extension for 45 s, and final extension for 10 min. Each reaction mix (25 µl) contained 50 ng of genomic DNA, 1× PCR buffer, 1.5 mM MgCl_2_, 200 µM dNTPs, 10 pmol each of sense and antisense primers (Table 1) and 1U of Taq DNA polymerase (Fermentas, Glen Burnie, MD). Thermal cycling was performed at suitable conditions using a VERITI 96 well thermal cycler (Applied Biosystems, Foster City, CA).

**Table 1:** List of primers used for PCR amplification of lens specific genes.

**Primer**	**Exon**	**Primer sequence 5’->3’**	**Product size (bp)**
*CRYAA-1F*	1	CTCCAGGTCCCCGTGGTACCA	254
*CRYAA-1R*		GCGAGGAGAGGCCAGCACCAC	
*CRYAA-2F*	2	CTGTCTCTGCCAACCCCAGCAG	223
*CRYAA-2R*		CCCCTGTCCCACCTCTCAGTGCC	
*CRYAA-3F*	3	GGCAGCTTCTCTGGCATGGGG	312
*CRYAA-3R*		GGGGAGCCAGCCGAGGCAATG	
*CRYBB2-1F*	1	TCTGTGGGCATTTGCTGACCC	292
*CRYBB2-1R*		GCTAACAGCATTGAAGTCTCTGCCC	
*CRYBB2-2F*	2	GACCCCACAGCTCTGGGACAGTC	393
*CRYBB2-2R*		GGAGGGACTTTCAGTATCAGCTCCAAC	
*CRYBB2-3F*	3	CACGGCTGCTTATAGCCAGAGCC	449
*CRYBB2-3R*		TCTATCTGACTGCAAAGCATGAATTATCTCC	
*CRYBB2-4F*	4	GCTTTGGGCACAGCGATGTTCTG	744
*CRYBB2-4R*		GGCCCCTTCCTGGTCCCCA	
*CRYBB2-5F*	5	AGTGGTCATAGACACGTAGTGGGTGCAC	706
*CRYBB2-5R*		CTGTTCCCAAACTTAGGGACACACGC	
*CRYBB2-6F*	6	CCCCTCGTTCACCCTCCCATCA	506
*CRYBB2-6R*		CACTGTGTCCAAGGTCACACAGCTAAGC	
*CRYGA-2F*	1&2	AGGTCCCTTTTGTGTTGTTTTTGCC	462
*CRYGA-2R*		CATGAGGAATTATACGGCAGGATTGG	
*CRYGA-3F*	3	CAGACCAGCTCGCACAAGTTAAGG	353
*CRYGA-3R*		AAGAGCCACTTAGTGCAGGGAACACAAC	
*CRYGB-2F*	1&2	TGCAAATCCCCTACTCACCAAAATGG	518
*CRYGB-2R*		AAAAAGATGGAAGGCAAAGACAGAGCC	
*CRYGB-3F*	3	TTTGTTTACTCTTGCGTTTTCTGTCTGCC	410
*CRYGB-3R*		GAAAGAAAGACAGGGCTCTACTAGTGCC	
*CRYGC-2F*	1&2	TGCATAAAATCCCCTTACCGCTGAG	522
*CRYGC-2R*		ACTCTGGCGGCATGATGGAAATC	
*CRYGC-3F*	3	AGACTCATTTGCTTTTTTCCATCCTTCTTTC	407
*CRYGC-3R*		GAAAGAATGACAGAAGTCAGCAATTGCC	
*CRYGD-2F*	1&2	GCAGCCCCACCCGCTCA	599
*CRYGD-2R*		GGGTAATACTTTGCTTATGTGGGGAG	
*CRYGD-3F*	3	TGCTTTTCTTCTCTTTTTATTTCTGGGTCC	400
*CRYGD-3R*		AGTAAAGAAAGACACAAGCAAATCAGTGCC	
*GJA8-L1F*	1	CGGGGCCTTCTTTGTTCTCTAGTCC	877
*GJA8-L1R*		AGGCCCAGGTGGCTCAACTCC	
*GJA8-L2F*	1	CAGCCGGTGGCCCTGCC	907
*GJA8-L2R*		GTTGCCTGGAGTGCACTGCCC	
*GJA3-1aF*	1	CTGCGATGCCTGTCCTGTGG	539
*GJA3-1aR*		TTGTCCTGCGGTGGCTCCTT	
*GJA3-1bF*	1	CGCCCACCCTCATCTACCT	549
*GJA3-1bR*		GTGGGAACCCGATGGCAAC	
*GJA3-1cF*	1	AGCTCAAGCAGGGCGTGACC	542
*GJA3-1cR*		CAAGGGCGGCTGGTGCATCT	
*GJA3-1dF*	1	CCCCGGCGCTCAAGGCTTAC	545
*GJA3-1dR*		AACCCTTGTCCCCGCCACCC	
*PAX6-1F*	1	CTCATTTCCCGCTCTGGTTC	300
*PAX6-1R*		AAGAGTGTGGGTGAGGAAGT	
*PAX6-2F*	2	CACACTCTTTATCTCTCACTCTCCAGCC	300
*PAX6-2R*		AATAAAGCGAGAAAGAAGCGGAC	
*PAX6-3F*	3	TCAGAGAGCCCATCGACGTAT	300
*PAX6-3R*		CTGTTTGTGGGTTTTGAGCC	
*PAX6-4F*	4	TTGGGAGTTCAGGCCTACCT	153
*PAX6-4R*		GAAGTCCCAGAAAGACCAGA	
*PAX6-5F*	5	CCTCTTCACTCTGCTCTCTT	257
*PAX6-5R*		ATGAAGAGAGGGCGTTGAGA	
*PAX6-5aF*	5a	TGAAAGTATCATCATATTTGTAG	237
*PAX6-5aR*		GGGAAGTGGACAGAAAACCA	
*PAX6-6F*	6	GTGGTTTTCTGTCCACTTCC	299
*PAX6-6R*		AGGAGAGAGCATTGGGCTTA	
*PAX6-7F*	7	CAGGAGACACTACCATTTGG	252
*PAX6-7R*		ATGCACATATGGAGAGCTGC	
*PAX6-8F*	8	GGGAATGTTTTGGTGAGGCT	371
*PAX6-8R*		CAAAGGGCCCTGGCTAAATT	
*PAX6-9F*	9	GTAGTTCTGGCACAATATGG	206
*PAX6-9R*		GTACTCTGTACAAGCACCTC	
*PAX6-10F*	10	GTAGACACAGTGCTAACCTG	243
*PAX6-10R*		CCCGGAGCAAACAGGTTTAA	
*PAX6-11F*	11	TTAAACCTGTTTGCTCCGGG	208
*PAX6-11R*		TTATGCAGGCCACCACCAGC	
*PAX6-12F*	12	GCTGTGTGATGTGTTCCTCA	300
*PAX6-12R*		TGCAGCCTGCAGAAACAGTG	
*PAX6-13F*	13	CATGTCTGTTTCTCAAAGGGA	957
*PAX6-13R*		GAACAATTAACTTTTGCTGGCC	

One affected member per family was screened for mutations in *CRYAA*, *CRYBB2*, *CRYGA* >*D*, *GJA3*, *GJA8*, and *PAX6* by SSCP analysis of PCR products. PCR products were purified by QIA quick purification kit (Qiagen, Hilden, Germany). The amplicons were mixed with SSCP denaturation solution (95% formamide, 10 mM NaOH, 0.25% bromophenol blue, 0.25% Xylene Cyanol- all reagents from Sigma-Aldrich, St. Louis, MO), denatured at 95 °C for 5 min and snap cooled on ice. Denatured samples were subjected to SSCP using DCode^TM^ Universal Mutation Detection System (Bio**-**Rad Laboratories, Hercules, CA). The denatured samples were then electrophoresed at 400 V for 16 h at RT as well as at 12 °C on 8%–10% polyacrylamide gels. The gels were silver stained for visualization of bands [18]. Samples showing variation in mobility as band shifts were further processed for sequencing.

### Restriction Fragment Length Polymorphism (RFLP)

RFLP analysis was performed using AluI restriction enzyme (New England Biolabs Ltd., NEB, Hitchin, Herts, UK) as per the manufacturer’s instructions in 10 µl volumes. The products were resolved on 2% agarose gel. The presence or absence of the AluI restriction site was checked in other family members and in 100 unrelated controls.

### DNA sequencing and structure prediction

Samples showing mobility shift were subjected to direct sequencing using Big Dye terminator chemistry on an ABI 3130 genetic analyzer (Applied Biosystems). Sequencing reaction was run through a program which included 25 cycles of denaturation (96 °C for 10 s), annealing (50 °C for 5 s), and extension (60 °C for 4 min). Sequence data were analyzed by standard software and alignments done by ClustalW or BLAST (GenBank NM_006891). The prediction of protein structure was made by using PDB deep view or PyMOL programs.

## Results

### Clinical findings

**Figure 1:**
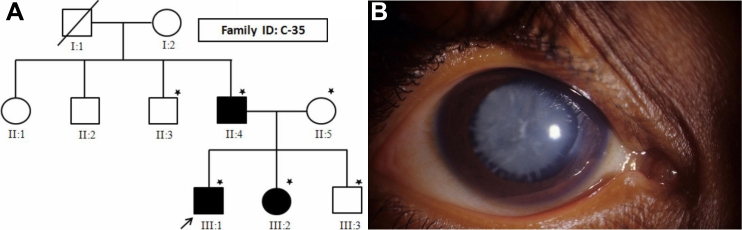
Pedigree of the C-35 family. **A**: The juvenile cataract affected family shows autosomal dominant mode of transmission with three affected individuals (II:4, III:1, and III:2). The arrow indicated the index case and the asterisk indicates members involved in the study. **B**: Slit lamp image of an individual with anterior polar coronary cataract phenotype is shown in the figure along with underlying anterior cortex. This is an example from CAT-35 family for a patient (proband III:1).

The study was performed on patients with familial nonsyndromic bilateral childhood cataract. During the period (March 2004 to April 2009) forty eight subjects from 17 families and 148 isolated cases with childhood cataract were evaluated. Among these, zonular cataract was most frequent (46%), followed by total (13%) posterior sub capsular (10%), and nuclear cataracts (8%). Blue dot, sutural, and membranous cataracts were also recorded. In family C-35 (Figure 1A) the proband (III:1) underwent a surgery for cataract removal at the age of 11 years. Right eye showed the phenotype as anterior polar with coronary cataract and left eye with only anterior polar cataract with a progressive loss of vision. Other ocular examination showed normal ocular movements with clear cornea. Visual acuity was found to be 6/60 (RE) and 6/36 (LE). The proband’s younger sister (III:2) of age 9 years presented anterior polar cataract of progressive nature in both eyes. Visual acuity was 6/24 in both the eyes. The proband’s father (II:4) showed anterior polar cataract in both eyes. The youngest sister (III:3) of the proband and the mother when examined for visual acuity have normal vision with clear cornea. A typical example of a slit lamp image of patient III:1 (proband in family CAT-35) is shown in Figure 1B.

### Mutation analysis

All exons and intron/exon boundaries and flanking sequences of the candidate genes, *CRYAA*, *CRYBB2*, *CRYGA*>*D*, *GJA3*, *GJA8*, and *PAX6* were screened by PCR-SSCP, RFLP, and DNA sequencing methods for detection of mutations. Consensus exon/intron boundaries in *CRYAA*, *CRYBB2*, *CRYGA*>*D, GJA3, GJA8*, and *PAX6* were verified by gene-specific primers designed to anneal to intronic sequence flanking exon boundaries.

**Figure 2:**
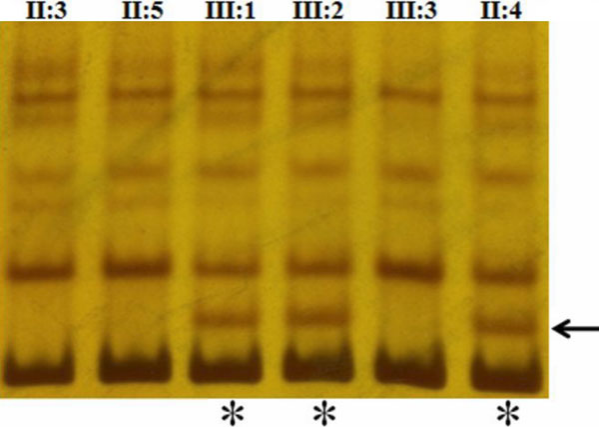
PAGE showing differential migration of affected samples by the SSCP method. The affected individuals (III:1, III:2, and II:4) showed differential banding pattern with an extra band on 8% PAGE and unaffected (II:3, II:5, and III:3) showed normal banding pattern. Arrow indicated the extra band and asterisk indicate the lanes showing differential migration.

We have identified a sequence alteration in exon 2 of *CRYGD* in a proband (C-35) diagnosed to have anterior polar and coronary cataract using SSCP (Figure 2). The samples showing the differential migration patterns were subjected to DNA sequencing. Sequence analysis of the three exons and immediate flanking regions in three affected family members (II:4, III:1, and III:2) using specific primers detected a heterozygous C>A transversion in exon 2 resulting in a missense substitution of arginine to serine at codon 77 (R77S) which was not present in unaffected family member (II:3, II:5, and III:3; Figure 3). The observed sequence variation was also confirmed through sequencing with the reverse primer in all the family members.

**Figure 3:**
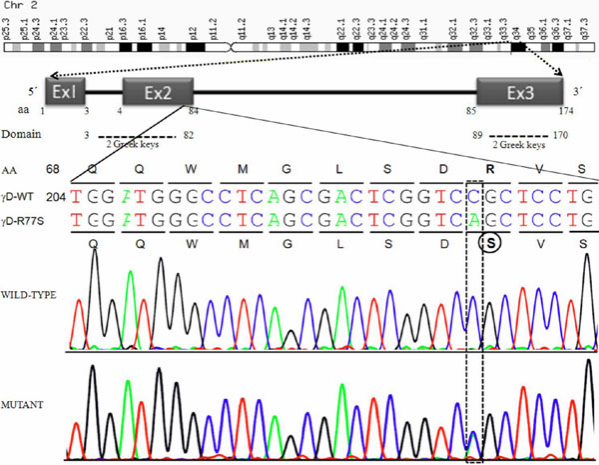
Genomic organization showing the region of R77S mutation. Electropherogram showing C>A heterozygous transversion in exon 2 *CRYGD*. The comparison of wild type and mutant sequences show c.229C>A substitution changes amino acid arginine to serine at position 77 (first amino acid is methionine). The dotted block shows the site of mutation and circle depicts the altered amino acid.

**Figure 4:**
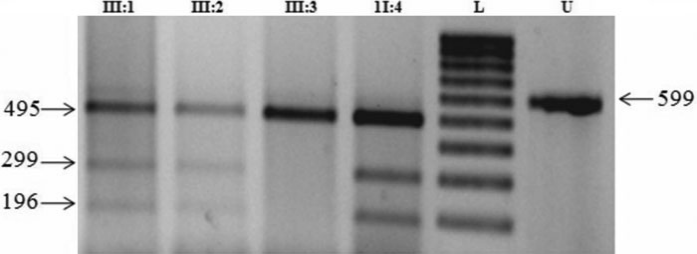
Restriction fragment length analysis of *CRYGD* exon 2. The mutation shows the gain of an Alu1 site (AGkCT) that co-segregated with affected individuals heterozygous for the C229A alteration (299,196, 69, and 35 bp), but not with unaffected individuals (495, 69, and 35 bp). L=100bp DNA ladder, U=undigested. Only one wild-type (III:3) is shown in the figure.

**Figure 5:**
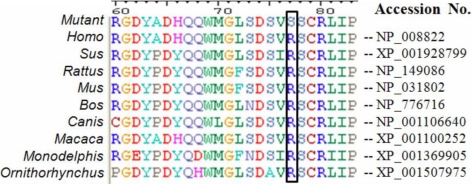
Multiple sequence alignment of γD-crystallin protein in different species. Sequence alignment showing the phylogenetic conservation of arginine at amino acid position 77. The mutant sequence indicates the sequence with the mutation detected in C-35 family. Only 60 to 83 amino acids are shown in the alignment.

This single-nucleotide change created an additional Alu1 restriction site in exon 2 of *CRYGD*. This Alu1 site (229AgkCT) co-segregated with affected individuals (II:4, III:1, and III:2) heterozygous for the R77S alteration (299, 196, 69, and 35 bp), but not in unaffected individuals (II:3, II:5; and III:3; 495, 69, and 35 bp) thus confirming the observed sequence variation and its segregation (Figure 4). We excluded this variation R77S of *CRYGD* as a single-nucleotide polymorphism in a panel of 100 normal unrelated subjects of the same ethnicity. Alignment of amino acid sequences of *CRYGD* as per the Entrez Protein database revealed that arginine at codon position 77 is phylogenetically conserved across species (Figure 5).

Taken overall, the co-segregation of R77S was seen only in affected members of the pedigree (C-35) and its absence in 200 normal chromosomes strongly suggest that the non-conservative R77S substitution might be a causative mutation rather than a benign polymorphism to be associated with the disease.

Sequencing of *CRYGD* gene in three affected (II:4, III:1, and III:2) and three unaffected individuals (II:3, II:5, and III:3) of C-35 family members showed four SNPs of which three (NG_008039.1:g.5277T>C; Y17Y; rs2242074, NG_008039.1:g.7677A>G; R95R; rs2305430, and NG_008039.1:g.7929T>C; rs2305429; 5′UTR) were reported earlier and a novel SNP E18F (NG_008039.1:g.5278G>A) was also observed. The inheritance of these five variations as a haplogroup in parents was analyzed (Table 2). It was found that the T-G-A-G-C haplogroup segregated with affected individuals showing transmission through paternal lineage.

**Table 2:** Haplotype analysis of SNPs/mutation found in *CRYGD* gene in C-35 family members.

** **	**Genotype**				
**SNPs (Mutation/polymorphism)**	**Father (Affected)**	**Mother**	**Son (Affected)**	**Daughter (Affected)**	**Son**
NG_008039.1 :g.5277T>C; Y17Y	CT	CC	CT	CT	CC
NG_008039.1 :g.5278G>A; E18F	GG	GA	GG	GG	GA
NG_008039.1 :g.5455C>A; R77S	CA	CC	CA	CA	CC
NG_008039.1 :g.7677A>G; R95R	GG	GA	GG	GG	GA
NG_008039.1 :g.7929T>C;-------	CC	CT	CC	CC	CT

Haplotypes involving the alleles at the SNP loci listed above, derived from pedigree of the family (Figure 1A). Haplotype analysis shows the inheritance of T-G-A-G-C haplotype segregating with affected individuals. SNP loci are given in the order in which they occur in the reference sequence of the genomic contig.

### Prediction of mutational change on protein properties

**Figure 6:**
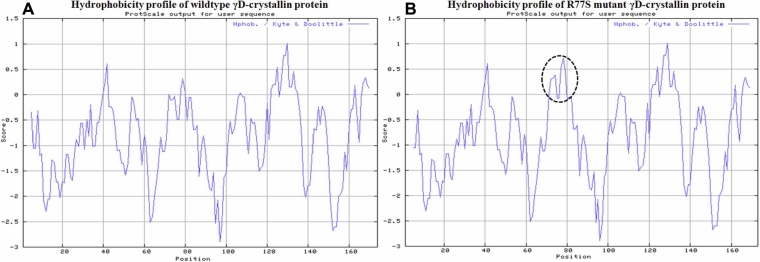
Hydrophobicity profile of wild-type and R77S mutant γD-crystallin protein. Dotted circle represent the shift in the hydrophobicity around the mutant site. The prediction was done by ProtScale program at Expasy server.

Both normal and mutant proteins were analyzed for their structure. The R77S (in the processed protein) is situated in second Greek key motif in the linker region as the last amino acid before start of the next beta sheet. The isoelectric point (pI) was found to be almost same for both wild type (7.0) and mutant (6.58) proteins. Molecular weight of the mutant (20,669 Da) protein was similar to that of wild-type protein (20,738 Da). There was an increase in hydrophobicity at the mutant site and its neighborhood (Figure 6).

The prediction of structural differences between wild-type and mutant proteins was performed using PyMOL tool. In the 3D model of human CRYGD protein (PDB code 1HK0), it was observed that the arginine side chain being longer protrudes out and might interact with the glutamic acid at position 46 forming ionic bonds. In the mutant form serine having a shorter side chain and being surrounded by valine and serine do not maintain the strong bonding with glu46 which may unfold the protein from its original folding. Arginine has well spread electron density enabling for high solubility. Moreover, the increased surface area in the arginine may facilitate better interaction with solvents, thereby reducing the solubility of mutant protein with substitution of serine without much change in kinase activity.

## Discussion

Genes reported to cause cataract-specific mutations include those of the crystallins, cytoskeletal proteins, membrane proteins, transcription factors, glucosaminyl transferase 2 chromatin modifying protein-4B, and transmembrane protein 114 (TMEM114) [19]. Crystallin specific mutations account for 16.6% of inherited pediatric cataract in south India [20]. Among the reported genes, crystallins are of special interest because it encodes the major proportion of water soluble structural proteins of the lens fiber cells. In vertebrates, the α-, β-, and γ- crystallins are ubiquitous lens proteins. γ- Crystallin amounts for 25% of the total crystallins in the human lens. Both *CRYGC* and *CRYGD* are expressed at high concentrations in the fiber cells of the human embryonic lens which subsequently form the lens nucleus. The precise cellular micro-architecture and homeostasis are critical in maintaining the transparency of the lens. Alterations that impairs the proper solubility of lens proteins can lead to progressive protein aggregation that might act as light scattering centers in the lens [11].

Human γD-crystallin is a monomeric eye lens protein that must remain soluble throughout life for lens transparency. It is composed of two highly homologous beta-sheet domains which interact through interdomain side chain contacts forming two structurally distinct regions, a central hydrophobic cluster and peripheral residues. The specificity of domain interface interactions is likely important for preventing incorrect associations in the high protein concentrations of the lens nucleus [21]. Features of the interface between the two domains conserved among γ-crystallins are a central six-residue hydrophobic cluster, and two pairs of interacting residues flanking the cluster. In human γD-crystallin these pairs are Gln54/Gln143 and Arg79/Met147. It is suggested that these residues stabilize the native state by shielding the central hydrophobic cluster from solvent. In aged and cataractous lenses, glutamine and methionine side chains are among the residues covalently damaged. Such damage may generate partially unfolded, aggregation-prone conformations of human γD-crystallin that could be significant in cataract [22].

Many of the identified mutations in *CRYGD* seems to have been associated with autosomal dominant cataract phenotypes of congenital or juvenile nature. The eleven mutations identified so far occur as missense [10,15,23-34], nonsense [15,34-36], and as frameshift [37] to have been associated with diverse phenotypes (Table 3). Three of the truncation mutations and a frameshift mutation seem to be associated exclusively with nuclear phenotype and all are congenital [15,34-36]. The phenotype described here (C-35) is an anterior polar and coronary and the age at onset being within the first decade of life. As this mutation (R77S) involves the substitution of a highly basic and polar charged Arg for a less polar Ser residue it probably may not cause any major conformational change, as suggested earlier [32]. Along with the R77S mutation, four other variations as SNPs haplogroup show the transmission of T-G-A-G-C block from affected father to two affected children. The significance of this mutation, along with the prevalence of any specific haplotype, remains to be confirmed through analysis from a large number of familial cataracts. Predictions on structural changes in the mutant form of γD-crystallin using PyMOL reveals the shorter Ser side chain to establish weaker bonding with Glu46 that might result in unfolding. Furthermore, substitution with serine reduces the surface area of interaction with solvents thus hampering solubility of the mutant form as also revealed by increase in hydrophobicity (Figure 6). This may therefore be considered as the causative mutation underlying the cataract phenotype in the family (C-35) investigated. Interestingly four of the *CRYGD* mutant alleles reported earlier also involves the residue Arg at different codon positions viz., 15, 37, 59, and 140 . In our study, by sequence alignment, the observed variation involves an arginine residue at position 77 which is highly conserved in γD-crystallin across various other species. Among cataract specific *CRYGD* mutations, the Arg residue has been replaced either by cysteine [10,23], serine [28,29,32], Histidine [38], or for a stop codon [36]. Similar to the substitution in this study, the R37S mutation has been reported earlier to result in phenotypes with protein crystals in a Czech [28] and in a Chinese study [29]. Recently, in a Chinese family a corolliform cataract was found to be associated with an R15S allele of *CRYGD* [32]. It has been suggested that an increase in hydrophobicity and a putative phosphorylation site-mediated protein aggregation as the probable cause of opacification [32]. Both R15S and R37S results in congenital cataracts and fall within the I Greek key motif, while R77S falls within II Greek key motif and in association with a juvenile form of cataract in the C-35 family. Cataract-specific mutations involving the Arg residue has also been frequently reported in *CRYAA* [34,35]. This suggests that Arg residues are more critical toward maintaining the structural and functional integrity of proteins.

**Table 3:** Mutation spectrum of human *CRYGD* and cataract phenotypes in different childhood cataract families.

**Exon**	**Nucleotide**	**Amino acid**	**Inheritance**	**Phenotype**	**Ethnicity**	**Ref**
Ex2	c.43C>T	p.Arg15Cys (R15C)	AD	Punctate cataract, juvenile progressive	Caucasian	[10]
Ex2	c.43C>T	p.Arg15Cys (R15C)	AD	Coralliform/nuclear	Chinese	[23]
Ex2	c.43C>A	p.Arg15Ser (R15S)	AD	Coralliform	Chinese	[32]
Ex2	c.70C>A	p.Pro24Thr (P24T)	AD	Lamellar	Indian	[15]
Ex2	c.70C>A	p.Pro24Thr (P24T)	AD	Cerulean	Moroccan	[24]
Ex2	c.70C>A	p.Pro24Thr (P24T)	AD	Coral-shaped, coralliform	Caucasian	[25]
Ex2	c.70C>A	p.Pro24Thr (P24T)	AD	Flaky, silica-like nuclear cataract	Australian pedigrees of European ancestry	[26]
Ex2	c.70C>A	p.Pro24Thr (P24T)	AD	Fasciculiform	Chinese	[27]
Ex2	c.70C>A	p.Pro24Thr (P24T)	AD	Coralliform	Chinese	[32]
Ex2	c.70C>A	p.Pro24Thr (P24T)	AD	Cerulean and Coralliform	Saudi Arabian	[33]
Ex2	c.109C>A	p.Arg37Ser (R37S)	AD	with protein crystallization	Czech boy	[28]
Ex2	c.109C>A	p.Arg37Ser (R37S)	AD	Nuclear golden crystal	Chinese	[29]
Ex2	c.168C>G	p.Tyr56Stop (Y56X)	AD	Nuclear	Brazilian	[34]
Ex2	c.176G>A	p.Arg59His (R59H)	AD	Aculeiform	Macedonian	[38]
Ex2	c.181G>T	p.Gly61Cys (G61C)	AD	Coralliform	Chinese	[30]
Ex2	c.229C>A	p.Arg77Ser (R77S)	AD	Anterior polar, Coronary	Indian	This study
Ex3	c.320A>C	p.Glu107Ala (E107A)	AD	Nuclear	Hispanic	[31]
Ex3	c.403C>A	p.Tyr134Stop (Y134X)	AD	No data	Danish	[35]
Ex3	c.418C>T	p.Arg140Stop (R140X)	AD	Nuclear	Indian	[36]
Ex3	c.470G>A	p.Trp157Stop (W157X)	AD	Nuclear	Indian	[15]
Ex3	c.494delG	p.Gly165fs	AD	Nuclear	Chinese	[37]

In the 16 other families studied no putative mutation could be observed in the candidate genes screened which therefore makes it rather unlikely that the selected genes are involved in the cataract-forming process in these families. It prompts screening of other known candidate genes. This demonstrates that cataract need not arise only through point mutations but might be influenced also by many other factors, which may include unidentified modifier genes and other sequence variations. Detailed information about such factors and their precise role should enable one to understand the pathophysiology of cataracts and the biology of the lens in general.
